# Characterization and Genome Analysis of *Staphylococcus aureus* Podovirus CSA13 and Its Anti-Biofilm Capacity

**DOI:** 10.3390/v11010054

**Published:** 2019-01-12

**Authors:** Yoyeon Cha, Jihwan Chun, Bokyung Son, Sangryeol Ryu

**Affiliations:** 1 Department of Food and Animal Biotechnology, Seoul National University, Seoul 08826, Korea; dianacha2006@naver.com (Y.C.); jihwanchun@gmail.com (J.C.); sonbk0722@gmail.com (B.S.); 2 Department of Agricultural Biotechnology, Seoul National University, Seoul 08826, Korea; 3 Research Institute of Agriculture and Life Sciences, Seoul National University, Seoul 08826, Korea; 4 Center for Food and Bioconvergence, Seoul National University, Seoul 08826, Korea

**Keywords:** bacteriophage, *Staphylococcus aureus*, anti-biofilm, genome analysis

## Abstract

*Staphylococcus aureus* is one of the notable human pathogens that can be easily encountered in both dietary and clinical surroundings. Among various countermeasures, bacteriophage therapy is recognized as an alternative method for resolving the issue of antibiotic resistance. In the current study, bacteriophage CSA13 was isolated from a chicken, and subsequently, its morphology, physiology, and genomics were characterized. This *Podoviridae* phage displayed an extended host inhibition effect of up to 23 h of persistence. Its broad host spectrum included methicillin susceptible *S. aureus* (MSSA), methicillin resistant *S. aureus* (MRSA), local *S. aureus* isolates, as well as non-aureus staphylococci strains. Moreover, phage CSA13 could successfully remove over 78% and 93% of MSSA and MRSA biofilms in an experimental setting, respectively. Genomic analysis revealed a 17,034 bp chromosome containing 18 predicted open reading frames (ORFs) without tRNAs, representing a typical chromosomal structure of the staphylococcal *Podoviridae* family. The results presented here suggest that phage CSA13 can be applicable as an effective biocontrol agent against *S. aureus*.

## 1. Introduction

*Staphylococcus aureus* possesses a diverse array of armaments that contribute to life-threatening infections, such as skin abscesses, endocarditis, pneumonia, and toxic shock syndrome [1]. Its ability to form biofilm on biotic and abiotic surfaces is one of the main hurdles for the eradication of *S. aureus* in both clinical and food environments [2,3]. Moreover, the emergence of antibiotic-resistant isolates in clinical environments poses a serious threat to global human health.

To cope with such problems, bacteriophages (phages) have been considered as alternative antimicrobial agents for treating multidrug-resistant bacteria and biofilm-associated infections [4,5]. Virulent phages have been considered to be promising as potential therapeutic agents, as they are safe, highly specific, and capable of lysing and killing infected target bacterial cells [6,7]. Furthermore, some phages produce enzymes with polysaccharide depolymerase activity, which can degrade capsular polysaccharides and exopolysaccharides (EPS), such as polysaccharide intercellular adhesin (PIA), a major structural component of most biofilms [5,8,9,10]. Recently, genetic engineering endowed phages with enhanced abilities for biofilm disposal, such as expressing stronger hydrolases or interfering quorum sensing [11].

Taxonomically, the majority of *S. aureus* phages are double-stranded DNA phages belonging to the *Siphoviridae* family, and a smaller number of them belong to the *Podoviridae* and *Myoviridae* families, which are primarily virulent phages [12]. Each family can be distinguished based on genome size and morphology: *Podoviridae* (<20 kb), short and non-contractile tail; *Siphoviridae* (≈40 kb), icosahedral head, long, non-contractile, flexible, and thin tail; *Myoviridae* (>125 kb), icosahedral head, long, contractile, rigid, and thick tail [12]. Due to their strict diversity, the *S. aureus* phages of each family retain characteristic genome structures, and hence, an orthologous genetic element does not necessarily appear across all the families [12]. The absence of universal genes in phage genomes has resulted in the development of alternative approaches, such as those based on structural genes, phage proteome, or phage orthologous groups (POGs), to unveil the biodiversity of these phages [13,14].

In this study, a new staphylococcal phage, CSA13, was isolated from a chicken, and its characteristics in terms of physiology, genomics, and bioinformatics were investigated. Specifically, phage CSA13 exhibited outstanding anti-biofilm activity against both methicillin-resistant *S. aureus* (MRSA) and methicillin-susceptible *S. aureus* (MSSA). As far as we know, this is the first report describing the use of a *Podoviridae* phage as an anti-biofilm agent. Taking into account its broad host range, along with its anti-biofilm activity, phage CSA13 could be developed as a promising biocontrol agent in both clinical and food industry settings.

## 2. Materials and Methods

### 2.1. Bacteriophage Isolation and Propagation

Bacteriophage CSA13 was isolated from chickens by using the *S. aureus* clinical isolate strain FMB-1 as a bacterial host. Whole chicken meat was homogenized for 1 min with 1 L of Bolton broth, supplemented with the Bolton Broth Selective Supplement (10 mg Cefoperazone, 10 mg Trimethoprim, 10 mg Vancomycin, and 25 mg Cycloheximide). The sample was then incubated at 42 °C for 12 h in micro-aerobic conditions (6% O_2_, 10% CO_2_, 84% N_2_). After incubation, the broth was centrifuged at 10,000× *g* for 5 min and filtered to remove bacterial cells. Following centrifugation and filtration, 5 mL of the filtrate was mixed with 5 mL of 2× Tryptic soy broth (TSB) and sub-cultured with the host strain at 37 °C, with shaking at 220 rpm for 12 h. After incubation, the culture was centrifuged at 10,000× *g* for 10 min and filtered to exclude any bacterial cells. The presence of phage in the filtrate was confirmed by spotting 10 µL of ten-fold, serial-diluted filtrates on soft agar (TSB containing 0.4% agar) containing 100 µL of host *S. aureus* culture. The plates were incubated overnight at 37 °C, and formation of plaques was monitored. Single plaques were picked with a sterile tip and eluted in sodium chloride-magnesium sulfate (SM) buffer (50 mM Tris-HCl, pH 7.5, 100 mM NaCl, 8 mM MgSO_4_·7H_2_O). This purification step was repeated more than three times. For propagation of the phage, TSB was first inoculated with the host *S. aureus* strain and incubated at 37 °C, with shaking at 220 rpm for 1.5 h. Subsequently, the phage was added at a multiplicity of infection (MOI) of 1, followed by a 3 h incubation in the same conditions. To prepare the phage at a high titer, the propagated phages were precipitated with polyethylene glycol (PEG) 6000 and concentrated by ultracentrifugation using a CsCl density gradient [15].

### 2.2. Transmission Electron Microscopy (TEM) Analysis

*Staphylococcus* phage CSA13 was analyzed using transmission electron microscopy (TEM). The phage suspension was placed on a carbon-coated copper grid and negatively stained with 2% uranyl-acetate (pH 4.0). The sample was examined under an energy-filtering transmission electron microscope at an operating voltage of 120 kV [16]. Phage CSA13 was identified and classified according to the guidelines of the International Committee on Taxonomy of Viruses.

### 2.3. Determination of the Bacteriophage Antimicrobial Spectrum

The bacterial strains listed in Table 1 were incubated overnight at 37 °C. A hundred microliters of each bacterial culture was mixed with 5 mL of soft agar (TSB containing 0.4% agar) and overlaid on tryptic soy agar (TSA) plates. Subsequently, 10 µL of serially diluted phage CSA13 lysates (ten-fold, 10^12^ to 10^5^ plaque forming unit (PFU)/mL) were spotted onto the prepared plates and incubated at 37 °C for at least 6 h in order to obtain single plaques. After incubation, the infectivity could be determined based on the appearance of the spots: “C”, clear single plaques; “T”, turbid single plaques; “I”, inhibited growth without single plaques; “-“, no lysis nor growth inhibition.

### 2.4. Bacterial Inhibition Assay

50 mL of TSB was inoculated with the host *S. aureus* strain and incubated at 37 °C until the early exponential growth phase (2.6 × 10^8^ ± 0.2 × 10^8^ CFU/mL). The culture was then infected with the phage at an MOI of 1. The OD_600_ was measured each hour after phage infection for 23 h, with no measurements being taken between the 14th and 22nd hours [17]. An un-infected culture was used as a control. All the experiments were performed in triplicate.

### 2.5. One-Step Growth Curve Assay

A one-step growth curve analysis was performed as described previously [18]. Briefly, phage was mixed with the *S. aureus* host strain in the early exponential growth phase at an MOI of 0.001. After incubation at 25 °C for 10 min to allow for adsorption of the phage, it was centrifuged at 6000× *g* for 10 min. The pellet containing infected cells was re-suspended in 50 mL of fresh TSB and incubated at 37 °C, with shaking at 220 rpm. Two sets of samples were collected every 5 min for 1 h. To release intracellular phages and determine the eclipse period, CHCl_3_ was added to one of the samples. Subsequently, the titre of each sample was immediately assessed using the double-layer agar plate method, and the latent period, eclipse period, and burst size were analyzed. All the experiments were performed in triplicate.

### 2.6. Receptor Analysis

The nucleotide sequence of the putative receptor binding protein (RBP) of phage CSA13 was predicted by nucleotide BLAST [19] analysis with that of phage S13′ and S24-1, which is annotated as ORF16 in each genome. The amino acid sequence of putative RBP of CSA13 was aligned with that of S13′ and S24-1 by Clustal X (ver. 2.0.11) [20], and the coverage and identity were analyzed by GeneDoc (ver. 2.7.000) [21]. The potential receptor for CSA13 was predicted based on the receptors for S13′ and S24-1 [22,23]. To verify the predicted receptor, *S. aureus* RN4220 [24] was used (Table 1). We obtained a RN4220Δ*tagO* mutant, which lacks the peptidoglycan-anchored wall teichoic acid (WTA) [25], and its complemented strain, carrying the pRB474-*tagO* plasmid. This plasmid was constructed by sub-cloning the *tagO* gene into an *Escherichia coli*-*S. aureus* shuttle expression vector [26]. Afterwards, 10 μL of ten-fold diluted phage CSA13 lysate (10^12^ to 10^5^ PFU/mL) were spotted from top-left to bottom-right on soft agar (TSB containing 0.4% agar) starting with the highest titer, with the wild-type RN4220, RN4220Δ*tagO* mutant, and the *tagO*-complemented strain.

### 2.7. Biofilm Reduction Assay with Phage CSA13

Biofilm reduction assays were performed based on a previous study with some modifications [27]. Two different *S. aureus* strains—namely, *S. aureus* Newman and *S. aureus* CCARM 3793, incubated in TSB supplemented with 0.25% d-(+)-glucose (TSBg) were prepared. After cultures were grown, 1:100 dilutions were performed by adding 2 μL of a pure cell suspension to 198 μL of TSBg in each well of a 96-well polystyrene plate, while 200 μL of TSBg was added as a negative control. After incubating the microplate for 24 h at 37 °C, all wells were washed three times with phosphate buffered saline (PBS). Once the biofilms were washed, they were treated with either 200 μL of phage lysate (10^9^–10^11^ PFU/mL) in SM buffer (50 mM Tris-HCl, pH 7.5, 100 mM NaCl, 8 mM MgSO_4_·7H_2_O) or a buffer-only negative control. After statically incubating at 37 °C for 24 h, phage lysate was removed, and each well was washed once with PBS and stained with 1% crystal violet. Additional washing with PBS was done, followed by solubilization with 33% acetic acid. The absorbance of the obtained solution was measured at 570 nm, and the sessile biomass was presented as an A_570_ value.

### 2.8. Bacteriophage Genomic DNA Purification

Bacteriophage genomic DNA was purified as previously described [28]. Prior to purification, the phage lysates were treated with DNase and RNaseA at 37 °C for 1 h to remove bacterial nucleic acid contaminants. The phage lysates were then treated with lysis buffer containing 0.5 M EDTA, 10 mg/mL proteinase K, and 1% sodium dodecyl sulfate (SDS) for 1 h at 56 °C. Finally, an ethanol precipitation was performed, followed by a phenol-chloroform DNA extraction.

### 2.9. Full-Genome Sequencing of Phage CSA13 and Bioinformatics Analysis

Purified CSA13 phage genomic DNA was sequenced using a Genome Sequencer FLX titanium system (Roche, Mannheim, Germany) and assembled with the GS de novo assembler software (Roche) at Sanigen Inc., South Korea. Open reading frames (ORFs) were predicted using the FGENESB (http://www.softberry.com), Glimmer v3.02 [29] and GeneMarkS [30] software packages. The ORFs were annotated using the InterProScan [31] and BLASTP [32] programs. Sequence alignments of CSA13 and staphylococcal phages from the *Podoviridae*, *Myoviridae*, and *Siphoviridae* families were performed by ClustalW [33], using whole-genome DNA sequences. Complete genome sequences of each taxon were acquired from the NCBI (www.ncbi.nlm.nih.gov) database. Sequence relationships were inferred using the neighbor-joining method [34], and the phylogenetic tree was constructed using MEGA7.0.21 [35]. The bootstrap value derived from 5000 replicates was considered to represent the evolutionary history of the analyzed taxa [36]. The evolutionary distances were computed using the p-distance method [37]. Comparative genome analyses of CSA13 and other *Podoviridae* phages were conducted by progressiveMauve [38,39] and ACT (Artemis Comparison Tool) [40]. Complete genome sequences of 16 staphylococcal *Podoviridae* phages were retrieved from the NCBI database: SAP-2, EU136189; BP39, KM366100; S13′, AB626963; S24-1, AB626962; SLPW, KU992911; SCH1, KY000084; SCH111, KY000085; 44AHJD, AF513032; P68, AF513033; 66, AY954949; PSa3, HF937074; GRCS, KJ210330; vB_SauP_phiAGO1.3, MG766218; vB_SauP_phiAGO1.9, MG766219; Andhra, KY442063; St 134, KY471386. The minimum score cut-off value of ACT analysis was 20. The complete genome sequence of *S. aureus* phage CSA13 was deposited in GenBank under the accession number MH107118.

## 3. Results and Discussion

### 3.1. Isolation and Physiological Characteristics of S. aureus Phage CSA13

The *S. aureus*-infecting phage CSA13 was newly isolated from a chicken. This phage formed clear plaques with a halo against the *S. aureus* clinical isolate FMB-1 as a bacterial host strain.

**Figure 1:**
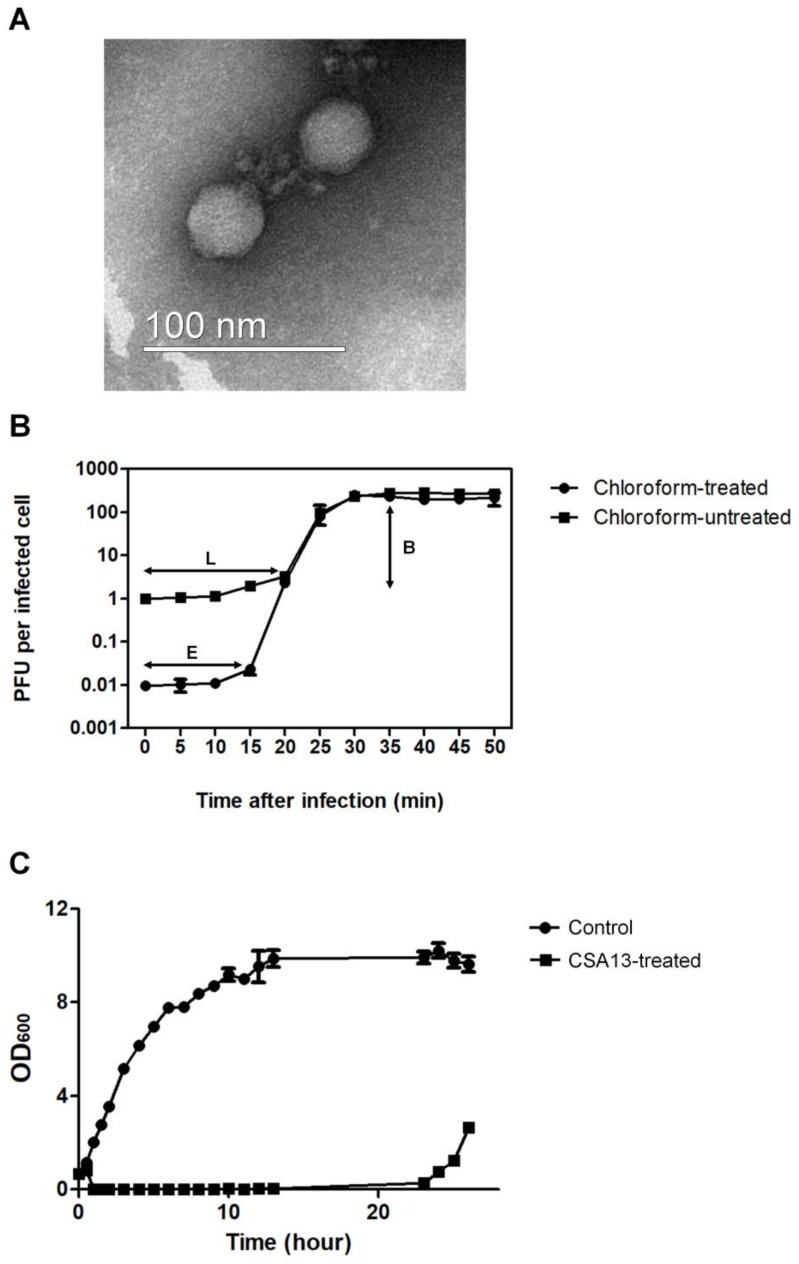
General characterizations of phage CSA13. (**A**) Electron micrograph of phage CSA13, which belongs to the *Podoviridae* family. The scale bar represents 100 nm. (**B**) One-step growth curve analysis of phage CSA13 on an exponential culture of *S. aureus* clinical isolate FMB-1 incubated in tryptic soy broth (TSB) medium at 37 °C, with shaking at 220 rpm. The culture of *S. aureus* clinical isolate FMB-1 was divided into two groups; chloroform-treated (●) or untreated (■). E, eclipse period; L, latent period; B, burst size. (**C**) Inhibition assays of *S. aureus* clinical isolate FMB-1 with phage CSA13 at 37 °C, with shaking at 220 rpm. Cells were prepared in two groups: control group without phage (●) or experimental group with phage CSA13, at an MOI of 1 (■). The data shown are the mean values from three independent measurements, and the error bars represent the standard deviations.

According to a previous study, the vast majority of known *S. aureus* phages belong to the *Siphoviridae* family [12], and only a small number, including phage SAP-2, belong to the *Podoviridae* family [41]. Transmission electron microscopy (TEM) revealed that phage CSA13 possesses a 40 nm icosahedral head with a short, non-contractile tail (Figure 1A). These results indicate that phage CSA13 is a member of the *Podoviridae* family, serogroup C, which features short-tailed viral particles. To determine the eclipse period, latent period, and burst size of phage CSA13, a one-step growth curve analysis was conducted with the host strain (Figure 1B). It was determined that the eclipse and latent periods were 15 min and 20 min, respectively. The burst size was about 230 PFU/infected cell, which is relatively higher than other prominent *S. aureus* phages. For example, among the *Myoviridae* family, phage K shows an average burst size of 60 PFU/infected cell [15] and phage Stau2 shows a burst size of approximately 100 PFU/infected cell [42]. These results indicated that phage CSA13 exhibits a high rate of phage production.

To determine the inhibition activity of phage CSA13, bacterial growth inhibition assays were performed (Figure 1C). The growth inhibition of *S. aureus* persisted for up to 23 h after infection with an MOI of 1.0, which is a stronger inhibitory effect than most other *S. aureus* phages, such as CS1 and DW2 [43], which show only 3 h of growth inhibition, or phage SA97, which showed up to 10 h inhibition with an MOI of 1.0 [44]. The above results demonstrate that phage CSA13 has a high replication rate and a strong inhibitory effect, which are essential properties for a phage to be used in phage therapy.

### 3.2. Receptor Analysis of Phage CSA13

**Figure 2:**
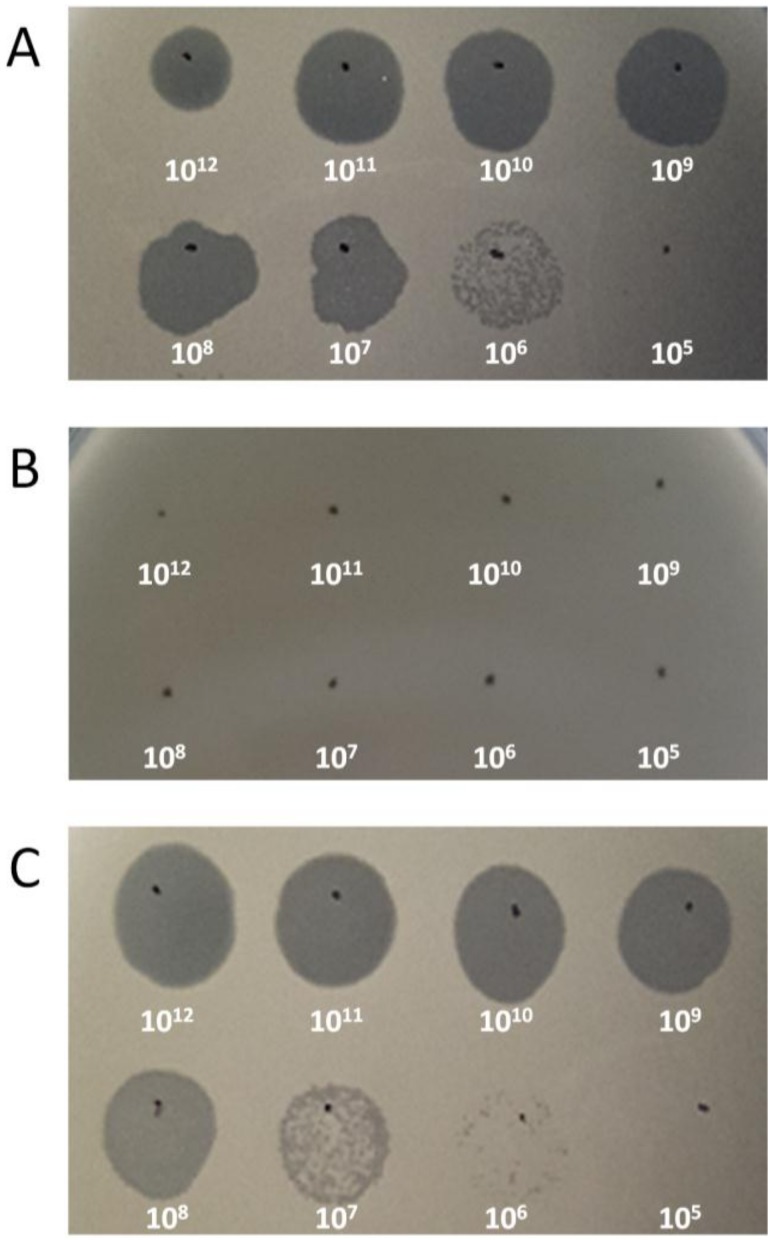
Wall teichoic acid (WTA)-dependent infection of *S. aureus* phage CSA13. (**A**) Phage CSA13 lysate was spotted onto lawns of wild-type RN4220, (**B**) *ΔtagO* mutant, and (**C**) *tagO*-complemented strains. Each number indicates the titer (PFU/mL) of phage CSA13 spotted on the plate. Plaque formation indicates successful adsorption and infection by phage CSA13.

It is known that peptidoglycan-anchored wall teichoic acids (WTA) of *S. aureus* serve as receptors for phage. For most cases, *S. aureus* synthesizes polyribitol phosphate WTA with substitutions such as α-O-*N*-acetylglucosamine (α-O-GlcNAc), β-O-*N*-acetylglucosamine (β-O-GlcNAc), or d-alanine [45]. In a previous study, it has been suggested that specific glycosylation pattern of WTA can prevent infection by *Podoviridae* [46]. For instance, *S. aureus Podoviridae* phage S13′ and S24-1 have been revealed to exhibit different binding capacity to WTAs harboring different glycosidic patterns. The putative receptor-binding protein (RBP) of S13′ requires β-O-GlcNAc for binding, while that of S24-1 can bind regardless of glycosidic bonds, which makes WTA itself the receptor for S24-1 [22,23]. BLAST analysis of the CSA13 genome revealed a gene-encoding putative RBP of CSA13, and its amino acid sequence was compared with that of S13′ and S24-1 (Figure S1). Putative RBP of CSA13 and S24-1 showed high sequence coverage (95%) and identity (97%), implying that CSA13 would recognize WTA as the receptor. Contrastingly, both RBP showed relatively low similarity to RBP of S13′: CSA13, 67% coverage, 79% identity; S24-1, 69% coverage, 80% identity. In accordance with the assumption, the *ΔtagO* mutant of *S. aureus* (RN4220*ΔtagO*) [47], which lacks WTA, was resistant to CSA13 (Figure 2B), and susceptibility to the phage was recovered by complementing the strain with the *tagO* gene (Figure 2C) [48].

### 3.3. Host Range of Phage CSA13

To determine the antimicrobial spectrum of phage CSA13, 28 strains of *S. aureus*, including 15 laboratory isolates, and eight additional Gram-positive strains were used, as shown in Table 1. The results indicate that CSA13 selectively infected *S. aureus* strains; 11 out of 13 *S. aureus*-type strains, including MRSA strains, and all 15 *S. aureus* local isolates were infected by CSA13. In addition, phage CSA13 could infect three other staphylococcal strains other than *S. aureus*, including *S. epidermis*, *S. hominis*, and *S. warneri*. The wide antimicrobial spectrum of phage CSA13 suggests its potential applicability as a biocontrol agent in clinical settings.

### 3.4. Biofilm Reduction Efficacy of Phage CSA13

**Figure 3:**
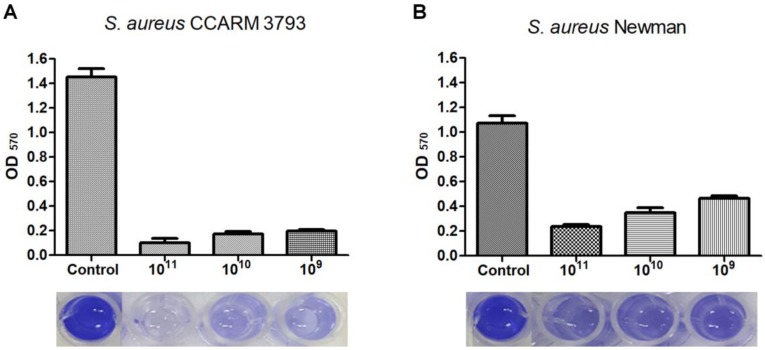
Removal of 24 h-old biofilm grown on a 96-well polystyrene microplate with phage CSA13. Biofilms of *S. aureus* CCARM 3793 (**A**) and Newman (**B**) were treated with three different PFUs of CSA13 for 24 h. Each column represents the mean of triplicate experiments, and error bars indicate the standard deviation.

The efficacy of phage CSA13 in reducing staphylococcal biofilm was examined by visual comparison based on the crystal violet staining method (Figure 3). In a 96-well polystyrene microplate, both *S. aureus* CCARM 3793 (MRSA) and Newman (MSSA) strains successfully formed biofilm in the presence of supplemented glucose. Biofilms formed by both *S. aureus* CCARM 3793 (Figure 3A) and *S. aureus* Newman (Figure 3B) were successfully removed by phage CSA13 in a PFU-dependent manner. Biofilm masses of *S. aureus* CCARM 3793 and *S. aureus* Newman were decreased by 86.5% and 56%, respectively, after a 24 h treatment with 10^9^ PFU/mL of CSA13. When the dosage was increased to 10^11^ PFU/mL, the biofilms were eradicated by 93.4% for CCARM 3793 and 78.5% for Newman, while the *S. aureus* CCARM 3793 strain displayed an approximately 40% stronger biofilm-forming capacity than *S. aureus* Newman. Considering that the antibiotic resistance of MRSA sacrifices virulence in exchange for an altered biofilm phenotype and elongated persistence [49,50], the significant biofilm reduction efficacy of CSA13 against *S. aureus* CCARM 3793 emphasizes its potential as an anti-biofilm candidate in clinical environments, which are vulnerable to colonization by the biofilm of MRSA strains [49,50,51].

Besides phage CSA13, several other *S. aureus* phages have also been tested for their anti-biofilm activity [5,52,53]. However, those previously studied *S. aureus* phages were not as effective as CSA13 at degrading staphylococcal biofilms. These results suggest that the anti-biofilm activity of phage CSA13 would be useful for the development of powerful biocontrol agents against both methicillin-susceptible and -resistant *S. aureus* strains.

### 3.5. Genome Analysis of Phage CSA13

**Figure 4:**
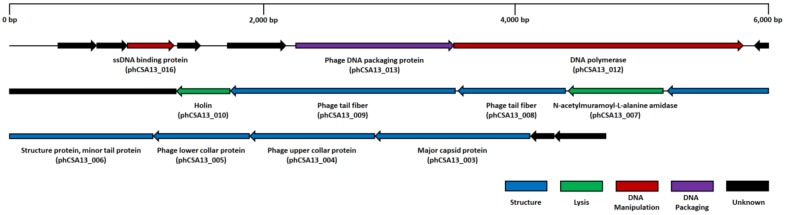
Genome map of phage CSA13. The arrangement of predicted ORFs on the CSA13 genome. Functional groups are categorized into colors.

Genomic features of phage CSA13 were revealed by whole genome sequencing. CSA13 is a double-stranded DNA (dsDNA) virus with a 17,034-base-pair-long linear chromosome harboring 234 bp-long inverted terminal repeat sequences at both ends. The average G + C content of the genome is 28.97%, and it contains 18 open reading frames (ORFs), but no tRNAs were predicted (Figure 4). Taken together with morphological traits, such genomic properties suggest that phage CSA13 is a member of the *Picovirinae* subfamily [54]. According to the clusters of the orthologous groups (COG) database, the ORFs were categorized into four functional groups: structural (major capsid protein, collar protein, tail fiber), lysis (*N*-acetylmuramoyl-l-alanine amidase, holin), DNA manipulation (DNA polymerase, ssDNA binding protein), and DNA packaging. Detailed information of predicted ORFs are specified in Table S1. Among the ORFs, two were predicted to exert lytic activity against the host. *N*-acetylmuramoyl-l-alanine amidase (phCSA13_007) is reported as one of five types of endolysin [55], which contribute to the hydrolysis of bacterial peptidoglycan. Endolysin is generally composed of two polypeptide domains: enzymatically active domains (EADs) at the N-terminal position, and cell wall binding domains (CBDs) at the C-terminal position [56]. The EAD and CBD of the predicted endolysin (phCSA13_007) were classified into cysteine, histidine-dependent amidohydrolases/peptidase (CHAP) (pfam05257), an amidase, and a SH3_5 (pfam08460) domain by BLASTP analysis [32]. On the chromosome, phCSA13_007 is located in between ORFs coding structural proteins, which is a distinctive feature of P68-like viruses [54].

### 3.6. Comparative Genome Analysis of Phage CSA13

**Figure 5:**
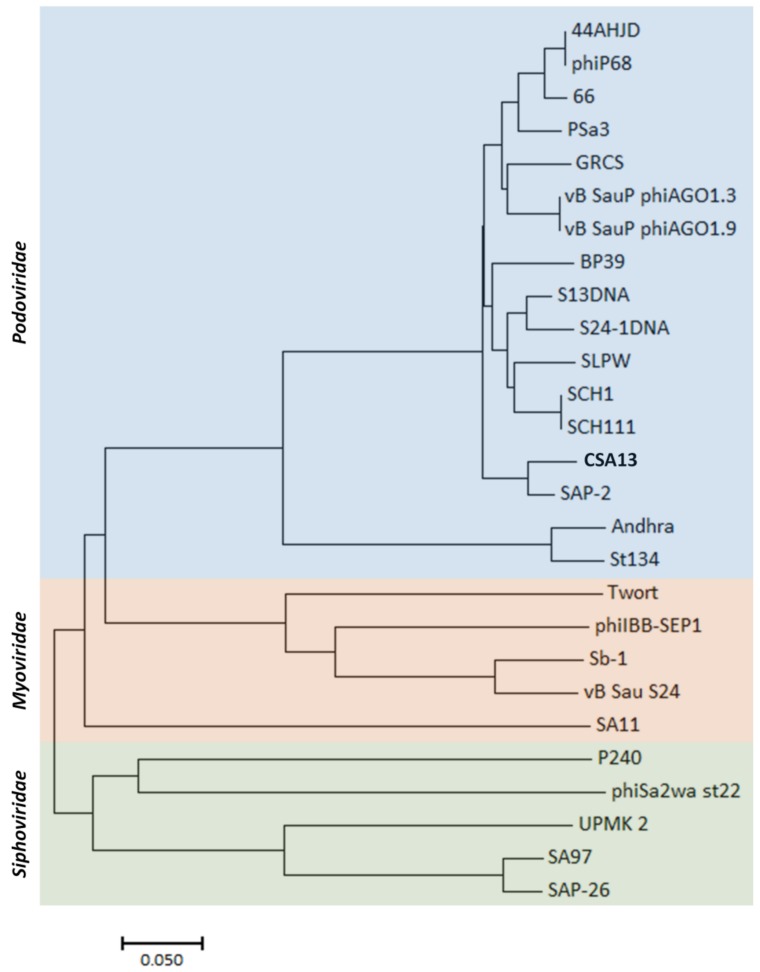
Phylogenetic tree of staphylococcal phages. Whole-genome sequences were aligned using ClustalW. The tree was constructed with neighbor-joining method, and the evolutionary distances were computed with the p-distance method by MEGA7 software.

In general, phylogenetic analysis of microbial entities relies on ribosomal RNA sequences [57]. In terms of bacteriophages, the absence of ribosomal sequence makes it difficult to classify them and measure their biodiversity. In previous studies, it was revealed that some phage proteins, such as terminase large subunits, are more conserved than others, implying that they could have a potential role as phylogenetic markers [58]. Despite the fact that some structural proteins were utilized as a basis to study phage phylogeny [59,60,61], due to their highly diverse nature, such genes could not serve as a universal marker for the biodiversity of phages. In fact, the coding genes for terminase are not predicted to be present in the genome of phage CSA13 and other *Podoviridae* phages. Hence, in this research, whole-genome sequencing of phage CSA13 and other *S. aureus Podoviridae* phages were analyzed together with *S. aureus Myoviridae* and *Siphoviridae* phage genomes (Figure 5). All of the *Podoviridae* phages are categorized into the *Picovirinae* subfamily, which has several unique features; those features include a small genome and similar numbers of genes. There are two genera in the *Picovirinae,* and *Staphylococcus* phages belong to the genus “P68virus” [54]. From the resulting tree, *Podoviridae* phages could be differentiated from other families, while a majority of *Podoviridae* phages were closely related to one another. Phage SAP-2 turned out to have the highest similarity to phage CSA13, even though most *Podoviridae* phages were not as diverse as those in the *Myoviridae* or *Siphoviridae* families.

**Figure 6:**
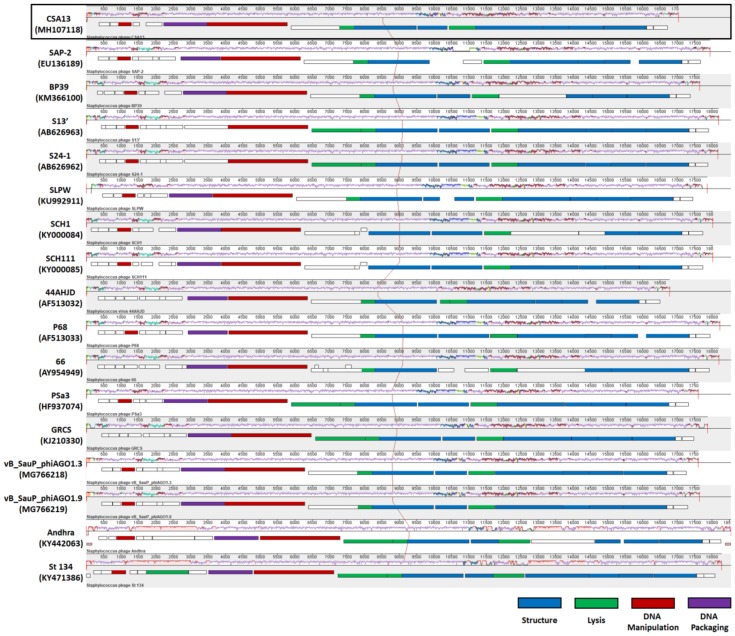
Multiple alignment of staphylococcal *Podoviridae* phage genomes by progressiveMauve. A total of 17 complete genomes were aligned; the genomes of phage CSA13 and phage 66 were annotated in an opposite direction to others; thus, the reverse complementary sequence was used. The GenBank accession number of each sequence is indicated. COG assignments for each ORF are represented in different colors, while the ORFs with unknown functions are depicted as blank boxes.

The relatively homologous nature of staphylococcal *Podoviridae* phages could also be observed from their genome structure (Figure 6). Due to their small genome (<20 kbp), there are less than 30 ORFs predicted on the genome of staphylococcal *Podoviridae* phages. Their linear genomes share a highly conserved structure and some very unique features, as described in a previous report [54]. The classical lysis cassette of lambdoid phages, which is composed of the lysin and holin genes [62,63], was not identified. Instead, endolysin, primarily amidases, appeared in between morphogenesis genes on the chromosome. In addition, among the structural genes, the gene encoding the capsid protein resided at the right end of the genome.

Despite a few variations, the overall similarity in genome structure could well explain the low diversity represented in the phylogeny.

**Figure 7:**
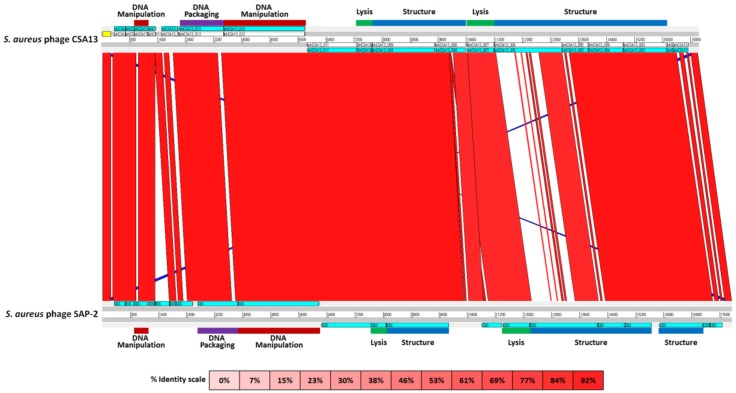
Comparative analysis of phage CSA13 and phage SAP-2 genomes by ACT. ORFs were designated with COG categories and corresponding color labels. The genome of phage CSA13 was annotated in an opposite direction; thus, the reverse complementary sequence was used. The color intensity of each band is proportional to the percent identity.

In comparison with the closest phage SAP-2, phage CSA13 exhibited strong sequence similarity (97% coverage with 97% identity) throughout the genome. As revealed in the ACT data, phCSA13_006, which was predicted to encode the “structure protein, minor tail protein”, displayed the most apparent difference when compared with phage SAP-2 (Figure 7). Low sequence similarity (68% coverage with 79% identity) between phCSA13_006 and ORF15 of SAP-2 was verified by amino acid sequence alignment analysis (Figure S1). Since phCSA13_006 is expected to serve as the receptor binding protein, a certain level of difference in amino acid sequence may contribute to a distinct host spectrum. The fact that ORF15 of SAP-2 was highly similar to ORF16 of S13′, which is unable to bind WTA-lacking β-O-GlcNAc, implies that the host range of SAP-2 is narrower than CSA13 [41]. In terms of amidase, the amino acid sequence comparison resulted in 88% identity with a coverage of 92% (Figure S2). Minor sequence diversity was observed in the CBD domain, which may also affect the variant host range between two species [64]. Examination of the properties of each amidase, for instance, the lysis activity or host spectrum, would be required for further research to build synergistic phage-mediated biocontrol strategies.

## 4. Conclusions

An integrated study upon newly discovered phage CSA13 revealed its physiological and genetic characteristics. Its inhibitory activity against bacterial hosts during interactions under various conditions, especially when host bacteria were developing biofilm, suggests that the phage itself can potentially serve as an effective biocontrol agent. Exploration of the genome uncovered its significantly conserved structure as a member of the staphylococcal *Podoviridae*. Despite the general similarity, phage CSA13 was distinguished from other *Podoviridae* phages, and further research would allow for insight into how such differences led to divergent phenotypes.

## Figures and Tables

**Table 1:** The antimicrobial spectrum of phage CSA13.

Bacterial Host	Lytic Activity *^a^*	Origin
**Staphylococcal strains**		
*S. aureus* RN4220	T	Laboratory strain
*S. aureus* Newman	I	Human
*S. aureus* ATCC 13301	C	N/A
*S. aureus* ATCC 23235	T	Food
*S. aureus* ATCC 33586	T	Human
*S. aureus* ATCC 33593	C	Human
*S. aureus* KCTC 1916	C	Human
*S. aureus* ATCC 6538	C	Human
*S. aureus* ATCC 29213	C	Human
*S. aureus* ATCC 12600	T	Human
MRSA CCARM 3793	C	Human
MRSA CCARM 3089	C	Human
MRSA CCARM 3090	I	Human
*S. haemolyticus* ATCC 29970	I	Human
*S. epidermidis* ATCC 35983	C	Human
*S. hominis* ATCC 27844	C	Human
*S. warneri* ATCC 10209	T	Antibiosis indicator for snake venoms
**Other Gram-positive bacteria**		
*Enterococcus faecalis* ATCC 29212	-	Human
*Bacillus cereus* ATCC 14579	-	Farmhouse
*Bacillus subtilis* ATCC 23857	-	N/A
*Listeria monocytogenes* ATCC 19114	-	Animal
**Laboratory isolates** (*S. aureus*)		
129	C	Animal
130	T	Animal
131	T	Animal
134	C	Animal
Clinical isolate 0055	C	Human
Clinical isolate 0136	C	Human
Clinical isolate 0154	T	Human
Clinical isolate 0212	T	Human
Clinical isolate 0600	C	Human
Clinical isolate-FMB_1	C	Cotton from hospital
77	T	Human
79	T	Human
80	C	Human
81	T	Human
82	T	Human

^*a*^ C, clear plaque; T, turbid plaque; I, inhibition zone; -, no lytic effect.

## Supplementary Materials

Supplementary materials can be found at http://www.mdpi.com/1999-4915/11/1/54/s1.
