# Supplementary material for: Characterization and Genome Analysis of Staphylococcus aureus Podovirus CSA13 and Its Anti-Biofilm Capacity
Source: Viruses. 2019 Jan 12;11(1):54. doi: 10.3390/v11010054 (PMC6356323; doi:10.3390/v11010054)
Supplement: Supplementary file 1 [file viruses-11-00054-s001.pdf]

Supplementary Data

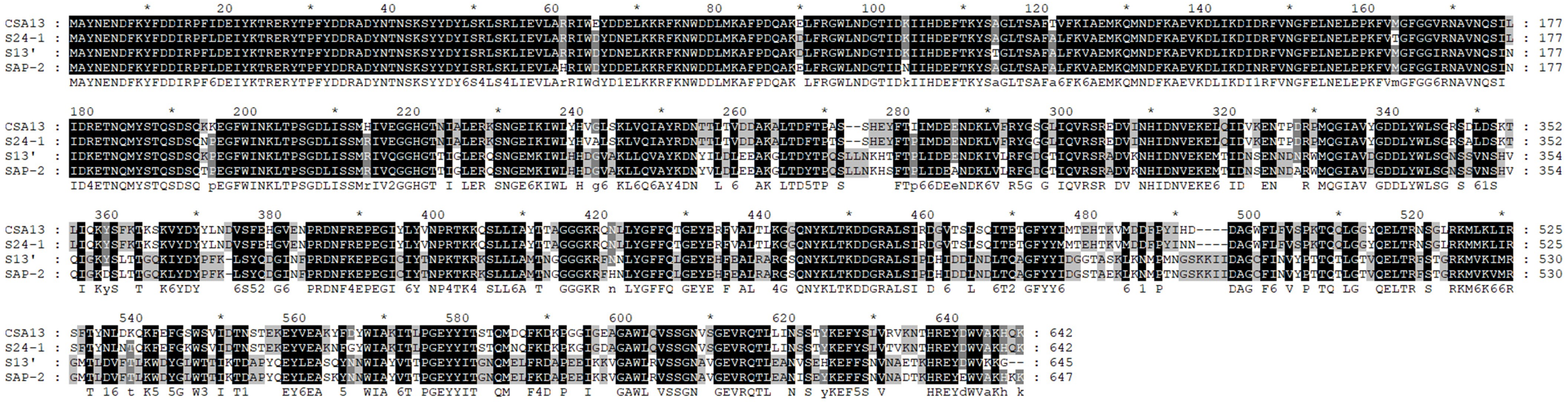

Figure S1. Sequence alignment of putative receptor binding protein (RBP)

The amino acid sequence of putative RBP from 4 different *S. aureus Podoviridae* phages were aligned using GeneDoc software. Alignment data between two sequences are as follows: CSA13 and S24-1, 95% coverage, 97% identity; CSA13 and S13', 67% coverage, 79% identity; CSA13 and SAP-2, 68% coverage, 79% identity; S24-1 and S13', 69% coverage, 80% identity; S24-1 and SAP-2, 69% coverage, 80% identity; S13' and SAP-2, 95% coverage, 97% identity. The proteins used for the multiple alignments have the following annotated locus_tag or GenBank accession numbers for putative RBP: CSA13, phCSA13_006; S24-1, BAL42303; S13', BAL42324; SAP-2, ABV44480. Conserved and identical residues are shaded in gray (dark gray, >70% conserved; light gray, >40% conserved) and black, respectively.

Table S1. Functional categories of predicted open reading frames (ORFs) in phage CSA13

Locus_tag [a]	Group	Subgroup	Function	BLASTP best match	Identity (%) [b]	Accession no. [c]
phCSA13_001	Unknown		Hypothetical protein	Hypothetical protein SAP2_gp20 [Staphylococcus phage SAP-2]	107/134 (80)	YP_001491545
phCSA13_002	Unknown		Hypothetical protein	Hypothetical protein SAP2_gp19 [Staphylococcus phage SAP-2]	59/60 (98)	YP_001491544
phCSA13_003	Structure	Head	Major capsid protein	Major head protein [Staphylococcus phage SAP-2]	405/408 (99)	YP_001491543
phCSA13_004	Structure	Head-tail	Phage upper collar protein	Putative upper collar protein [Staphylococcus phage GRCS]	321/327 (98)	YP_009004308
phCSA13_005	Structure	Head-tail	Phage lower collar protein	Lower collar protein [Staphylococcus phage SCH111]	239/251 (95)	APD20965
phCSA13_006	Structure	Tail	Structure protein, minor tail protein	Structure protein, minor tail protein [Staphylococcus phage S24-1]	610/642 (95)	BAL42303
phCSA13_007	Lysis		N-acetylmuramoyl-L-alanine amidase	CHAP domain-containing protein [*Staphylococcus aureus*]	230/249 (92)	WP_031863627
phCSA13_008	Structure	Tail	Phage tail fiber	Tail fibers protein [Staphylococcus phage SA4]	271/298 (91)	ASZ78265
phCSA13_009	Structure	Tail	Phage tail fiber	Major tail protein [Staphylococcus phage Pabna]	577/591 (98)	AYP28472
phCSA13_010	Lysis		Holin	Holin [Staphylococcus phage SAP-2]	132/140 (94)	YP_001491536
phCSA13_011	Unknown		Hypothetical protein	Hypothetical protein [Staphylococcus phage SA4]	463/475 (97)	ASZ78262
phCSA13_012	DNA manipulation		DNA polymerase	DNA polymerase [Staphylococcus phage SAP-2]	749/761 (98)	YP_001491534
phCSA13_013	DNA packaging		Phage DNA packaging protein	DNA packaging protein [Staphylococcus phage SCH1]	412/415 (99)	APD20934

phCSA13_014	Unknown	Hypothetical protein	Hypothetical protein SAP2_gp07 [Staphylococcus phage SAP-2]	149/153 (97)	YP_001491532
phCSA13_015	Unknown	Hypothetical protein	Hypothetical protein [Staphylococcus phage SLPW]	56/59 (95)	YP_009278557
phCSA13_016	DNA manipulation	ssDNA binding protein	Single stranded DNA-binding protein [Staphylococcus phage SLPW]	114/122 (93)	YP_009278556
phCSA13_017	Unknown	Hypothetical protein	Hypothetical protein SAP2_gp02 [Staphylococcus phage SAP-2]	74/78 (95)	YP_001491527
phCSA13_018	Unknown	Hypothetical protein	Hypothetical protein SAP2_gp01 [Staphylococcus phage SAP-2]	98/100 (98)	YP_001491526

a, Each predicted ORF of phage CSA13 was labeled with locus_tag; b, Amino acid sequence identity was calculated; c, GenBank accession number of ORF

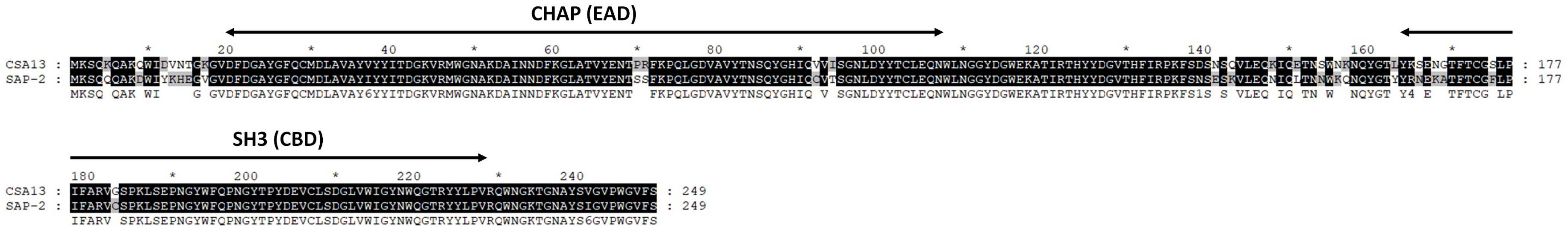

Figure S2. Sequence alignment of endolysin.

The amino acid sequence of endolysin from phage CSA13 and SAP-2 were aligned using GeneDoc software. Alignment data between two sequences are as follows: 88% coverage, 92% identity. The proteins used for the alignments have the following annotated locus_tag or GenBank accession numbers for endolysin: CSA13, phCSA13_007; SAP-2, ABV44479. Conserved and identical residues are shaded in gray (dark gray, >70% conserved; light gray, >40% conserved) and black, respectively.
